# Predicting Post-Dive Inert Gas Bubble Grades in Non-Decompression Scuba Diving with Air: Simplified Model for Enhanced Diver Safety

**DOI:** 10.1186/s40798-025-00832-x

**Published:** 2025-03-28

**Authors:** Andreas Fichtner, Björn Hannesen, Felix Stein, Benedikt Schrofner-Brunner, Thomas Pohl, Thomas Grab, Thea Koch, Tobias Fieback

**Affiliations:** 1 Medical Faculty C.-G.-Carus, TU Dresden, Dresden, Germany; 2 TU Bergakademie Freiberg, Freiberg, Germany; 3 Klinikum Region Hannover, Burgwedel, Germany; 4 Klinikum Chemnitz, Chemnitz, Germany; 5 Department of Marine Sciences, University of Gothenburg, Göteborg, Sweden

## Abstract

**Background:**

Even well-planned no-decompression dives can still produce inert gas bubbles that increase decompression sickness risk. A previously proposed formula for predicting post-dive bubble grades integrates individual factors (age, breathing gas consumption) with dive parameters (maximum depth, surface interval). This study aimed to confirm the formula’s validity in an independent dataset and to find out whether detailed dive profile data are of further relevance in predicting echocardiography-derived post-dive bubble grades. Additionally, we explored whether machine learning models leveraging detailed dive profile data could enhance predictive accuracy.

**Results:**

A total of 59 divers performed 359 no-decompression open-circuit air dives in freshwater and saltwater. Post-dive transthoracic echocardiography detected bubbles (Eftedal-Brubakk grade ≥ 1) in 29.8% of dives. Maximum depth, total dive time, air consumption, and age correlated significantly with observed bubble grades (r_s_=0.37, r_s_=0.16, r_s_=0.27, r_s_=0.13, respectively). The original prediction formula remained valid (r_s_=0.39) and adequately captured higher-grade dives. Spending additional time in shallow water after deep segments reduced bubble formation. Machine learning approaches based on typical dive computer data (e.g. dive profile) provided stronger predictions (r_s_=0.49).

**Conclusions:**

This study shows that maximum depth, age, surface interval and total breathing gas consumption are sufficient predictors of post-dive bubble load in no-decompression air dives. This allows divers to individually adopt bubble-reducing measures—such as resting, hydrating, and extending surface intervals—once alerted to a higher-risk class. Integrating the formula into dive computers may offer real-time, individualised risk guidance and help prevent decompression sickness despite following computer-derived profiles in recreational diving.

## Background

SCUBA diving has become increasingly accessible as both a recreational activity and a profession, yet the physiological consequences of diving—particularly the changes in ambient pressure—remain significant. During a dive using compressed air as breathing gas, the body absorbs inert gas (primarily nitrogen) at elevated partial pressures. Upon ascent, decreases in ambient pressure can lead to supersaturation of tissues and the formation of intravascular and extravascular gas bubbles [1–3]. In many cases, these bubbles are filtered out via the pulmonary circulation or remain asymptomatic, but they can nevertheless pose health risks if they form in excess or cross from the venous to the arterial system through anatomical shunts, such as a patent foramen ovale [4, 5].

Modern dive computers were developed to mitigate decompression sickness (DCS) by calculating inert gas uptake based on tissue compartments with distinct kinetics [6, 7]. These devices recommend ascent profiles and safety stops to reduce the likelihood of bubble formation [8]. However, dive computers tend to focus on “one-size-fits-all” algorithms without fully accounting for individual physiological factors like age, body composition, vascular characteristics, hydration state, and underlying health conditions [3, 9]. While these algorithms have significantly improved diving safety [10], unexplained—or “undeserved”—accidents continue to be reported among recreational and professional divers, with noted dependency on individual factors such as age [11–13]. This observation indicates that individual diver variability can play a meaningful role in decompression outcomes.

A key advance in understanding these individual differences has come from direct measurement of bubble loads in the vasculature, typically assessed via ultrasound or transthoracic echocardiography [8, 14]. The Eftedal-Brubakk (EB) scoring system is often used to quantify intravascular bubbles from 0 (no bubbles) to 5 (extensive bubbles), thereby providing an objective measure of decompression stress [1, 15]. Studies have shown that bubble formation is influenced by dive parameters such as maximum depth, total time, ascent rate, and type of breathing gas [7, 16]. Additionally, extended safety stops at shallow depths, hydration, rest, and conservative diving practices have been linked to fewer observed bubbles [8, 15]. Our previous study [8] tested for multiple regression of many individual factors (e.g. body composition, sex, smoking, exertion, mental stress, fluid loss, etc.) and extended dive-related factors (such as salinity, water temperature, etc.) and found no significant correlation of most individual factors to post-dive bubbling. The few correlated factors showed a maximum correlation of post-dive inert gas bubbling in the combination of the dive-related factor maximum depth, pre-existing inert gas load specified as surface interval up to 48 h, individual factor age and combined dive and individual factor air consumption. These four parameters, combined in a field formula, were sufficient to predict post-dive echocardiography-derived bubble grades. The parameter of air consumption reflects not only the dive profile (i.e. dive time and depth) but also individual factors such as physical and psychological exertion, experience, and body temperature regulation – all of which influence inert gas saturation and desaturation during a dive.

Despite improvements in basic decompression algorithms, divers continue to report mild neurological or musculoskeletal complaints—sometimes diagnosed as subclinical DCS [17]. The rising interest in applying machine learning and advanced statistical techniques [18] aims to refine risk models by incorporating not only dive-profile data (depth, time, ascent rate) but also individual physiological or behavioural factors (e.g., age, hydration status, or real-time heart rate variability). This approach has the potential to further reduce unexpected dive-related incidents by providing personalised feedback [3, 19].

### Aims of this Study and Hypotheses

1. Post-dive inert gas bubbling after sports Scuba diving using compressed air can be estimated on an individual basis using a combination of four diving and individual parameters, namely maximum depth, breathing gas consumption, surface interval, and age, as described in the previously proposed field formula [8].
2. Integrating detailed dive profile data enhances the predictive accuracy for post-dive bubble formation provided by the four core parameters.
3. Contribute to modern dive safety guidelines by identifying new approaches to individualised risk estimation, aiming to reduce the incidence of unprovoked and unexplained diving accidents through individual measures such as increased fluid intake and extended resting surface intervals.

## Methods

### Study Setup

After ethics committee approval, we examined 59 adult scuba divers (ages 19–64 years; 13 female and 46 male) across 18 days in 2022, yielding 359 dives. All participants were certified recreational sports divers performing single and repetitive dives in freshwater (Ammelshain, Senftenberger See, Germany) and saltwater (Sveta Marina, Adriatic Sea, Croatia). Every diver held a valid fit-to-dive certificate. Only dives on compressed air were included. A large portion of the divers were scientific divers in training [20]. Their dives involved tasks such as underwater measurement exercises and cartography but no heavy labour. Each diver carried a modern, fully functional dive computer.

Before each dive series, participants were measured and weighed, and written informed consent was obtained. After exiting the water, each diver walked approximately 150 m in full gear back to the dive base and then rested for 30 min. Standardised transthoracic echocardiography (Model GE logiq e, GE Healthcare, Chicago, USA) video loops were subsequently recorded. We logged biometric data (e.g. height, weight, smoking status, sex) and detailed dive profiles (collected in 5-minute intervals). Divers also reported their gas consumption, maximum depth, and any extraordinary occurrences during the dive—such as panic, discomfort, feeling cold, or dive computer alerts.

### Data Analysis

All statistical analyses were performed in R 4.4.1 (R Core Team, Vienna, Austria). We used Spearman’s rank correlation coefficients for the initial exploration of associations between bubble grades (0–5 on the Eftedal–Brubakk scale) and both diver-specific (e.g., age, BMI, smoking status, sex) and dive-specific (e.g., maximum depth, dive duration, air consumption) factors. This method did not require us to transform the data and could handle the ordinal nature of the bubble grades while accounting for potential non-linear relationships between variables [21].

To validate the prediction formula proposed by Fichtner et al. [8], we calculated predicted bubble grades using:

*EB Bubble grade = (age[y]/50 − surface interval[hrs]/150 + maxdepth[m]/45 + air consumption[barl]/4500)* ^*2*^

These predictions were then compared with observed bubble grades to assess accuracy.

For a deeper analysis of factors affecting post-dive bubble grade, we applied a linear mixed-effects model (lmer; Bates et al., 2015), with EB grade as the outcome, maximum depth and shallow-depth time as predictors, and diver ID as a random effect (1|Diver). This approach accounted for repeated measures from the same individuals. The response variable was log + 1-transformed to address the logarithmic nature of the bubble grade. The normality of residuals was inspected graphically using Q-Q plots [21].

Model Specification:

*log(EB Grade +1) ~ maximum depth [m] + time above 10 m depth after maximum depth [min] + interaction + (1|Diver)*.

We also employed Random Forest models [18, 22] to predict EB bubble grade using different sets of variables (Table 1). Each model was trained on 70% of the data and tested on the remaining 30%. Performance was assessed by Spearman’s rank correlation between predicted and observed bubble grades.

**Table 1:** Variable sets used in random forest models to predict EB bubble grades. Each set represents an incremental addition of person-specific and dive-specific parameters

Variable set	Variables
Person-Specific Variables	age, sex, BMI
Minimal Dive Data	maximum depth, dive time
Person-Specific + Dive Data	age, sex, BMI, maximum depth, dive time
Person-Specific + Dive Data + Air	age, sex, BMI, maximum depth, dive time, Air consumption
Dive Computer Data	maximum depth, dive time, time spent between 0 and 10 m, time spent between 10 and 20 m, time spent between 20 and 30 m, time spent between 30 and 40 m, time spent between 0 and 10 m after max depth, time spent between 10 and 20 m after max depth, time spent between 20 and 30 m after max depth, time spent between 30 and 40 m after max depth
All	All variables

EB: Eftedal–Brubakk, BMI: Body Mass Index (kg/m²), m: Meters

## Results

A total of 359 dives performed by 59 recreational scuba divers (13 females and 46 males) were analysed. Participants ranged in age from 18 to 63 years, with a mean of 38.2 years (SD = 13.9). Dive depths varied from 4 to 45 m, averaging 24.2 m (SD = 11.0), and dive durations ranged from 20 to 90 min, with a mean of 50.4 min (SD = 13.7).

**Fig. 1:**
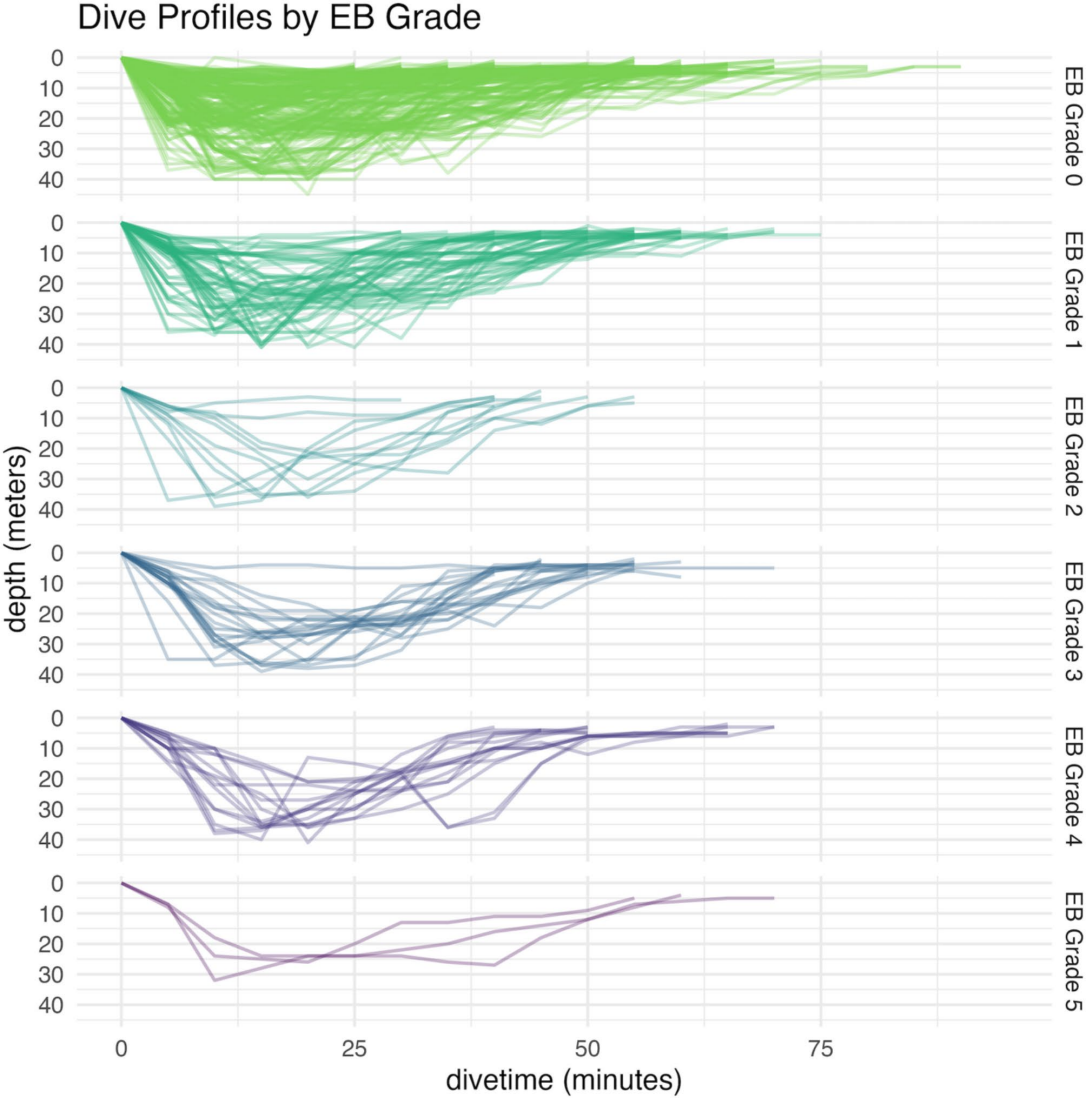
Diving profiles of all 359 dives faceted by bubble grade

Post-dive echo-derived bubble grades, assessed using the Eftedal–Brubakk (EB) scale, were distributed as follows: 252 dives (70.2%) had a grade of 0; 68 dives (18.9%) had a grade of 1; 11 dives (3.1%) had a grade of 2; 20 dives (5.6%) had a grade of 3; 15 dives (4.2%) had a grade of 4; and 3 dives (0.8%) had a grade of 5 (Fig. 1). In total, 107 dives (29.8%) exhibited post-dive bubbles (EB grade ≥ 1). None of the divers showed any symptoms of DCS. Figure 1 indicates that dives with a bubble grade of 0 were generally associated with shallower profiles and extended time in shallow water. In contrast, dives with bubble grades ≥ 2 tended to be deeper, with divers spending more time at greater depths.

Correlation analyses (Table 2) revealed that the strongest single predictor of post-dive bubble grade was maximum dive depth (r_s_ = 0.37, *p* < 0.001). This was followed by air consumption during the dive (r_s_ = 0.27, *p* < 0.001), total dive time (r_s_ = 0.16, *p* = 0.002), and age of the diver (r_s_ = 0.13, *p* = 0.011). No significant differences in bubble grades were observed between male and female divers (*p* > 0.05).

**Table 2:** Spearman correlation with bubble grade

Correlation with bubble grade	Spearman Correlation (*r*_s_)	*p* value
Age (y)	**0.13**	0.013
BMI (kg/m²)	-0.06	0.203
Air consumption (litre)	**0.27**	< 0.001
Total dive time (min)	**0.16**	0.002
Maximum depth (m)	**0.37**	< 0.001
First or second dive of the day (Y/N)	-0.04	0.449
Time between 0 and 10 m after max depth (min)	-0.09	0.071
Time between 10 and 20 m after max depth (min)	-0.07	0.252
Time between 20 and 30 m after max depth (min)	0.06	0.413
Time between 30 and 40 m after max depth (min)	0.06	0.704
Total time between 0 and 10 m (min)	**0.09**	0.093
Formula by Fichtner et al. 2021	**0.39**	< 0.001

**Fig. 2:**
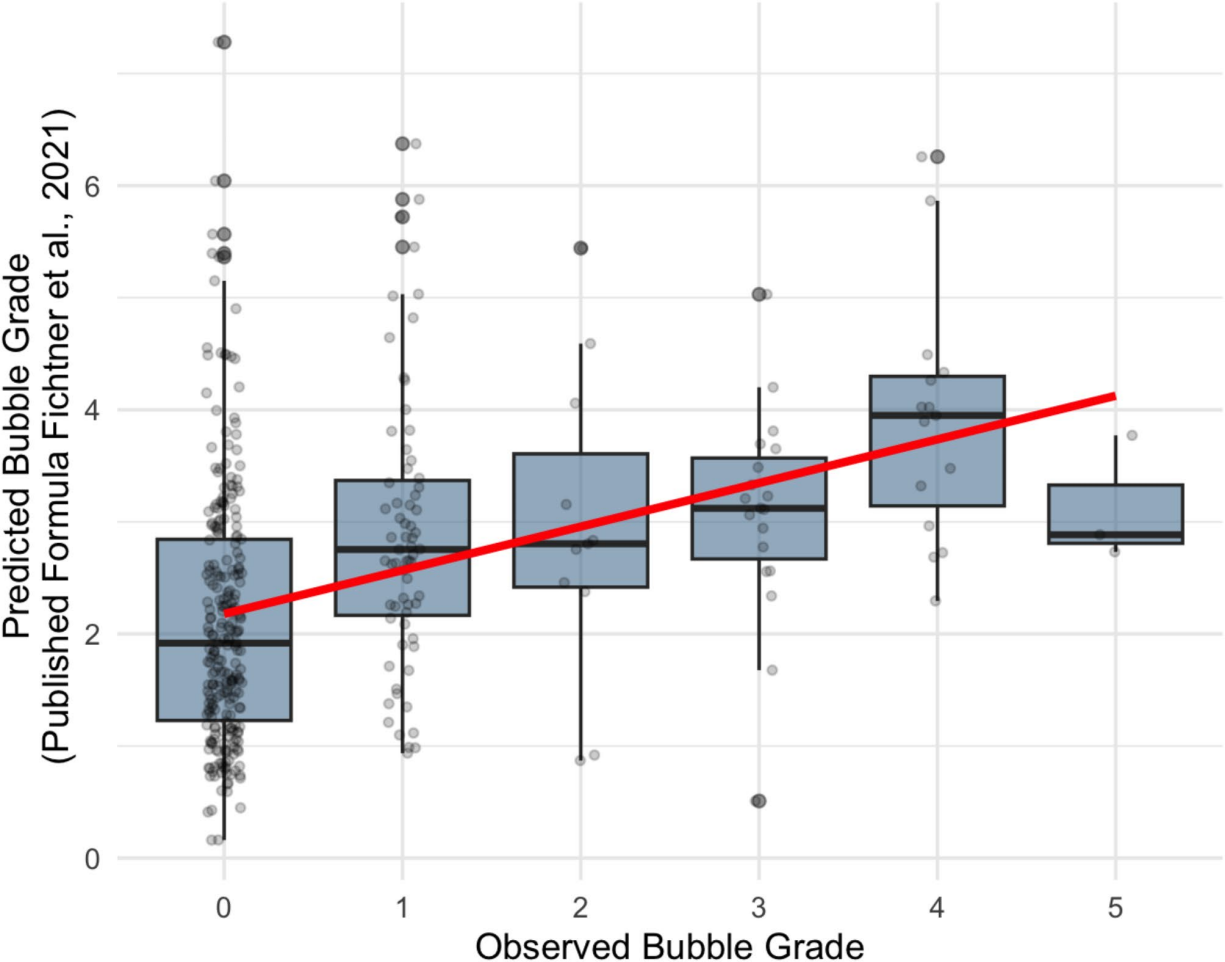
Predicted bubble grade and observed bubble grade using prediction formula by Fichtner et al. 2021

The predictive formula proposed by Fichtner et al. [8], which incorporates maximum depth, age, surface interval, and air consumption, demonstrated a slightly higher correlation with bubble grade (r_s_ = 0.39, *p* < 0.001) compared to maximum depth alone. This formula provides the advantage of estimating the EB grade with greater certainty and is optimised so as not to underestimate the risk of a high EB grade (Fig. 2).

**Fig. 3:**
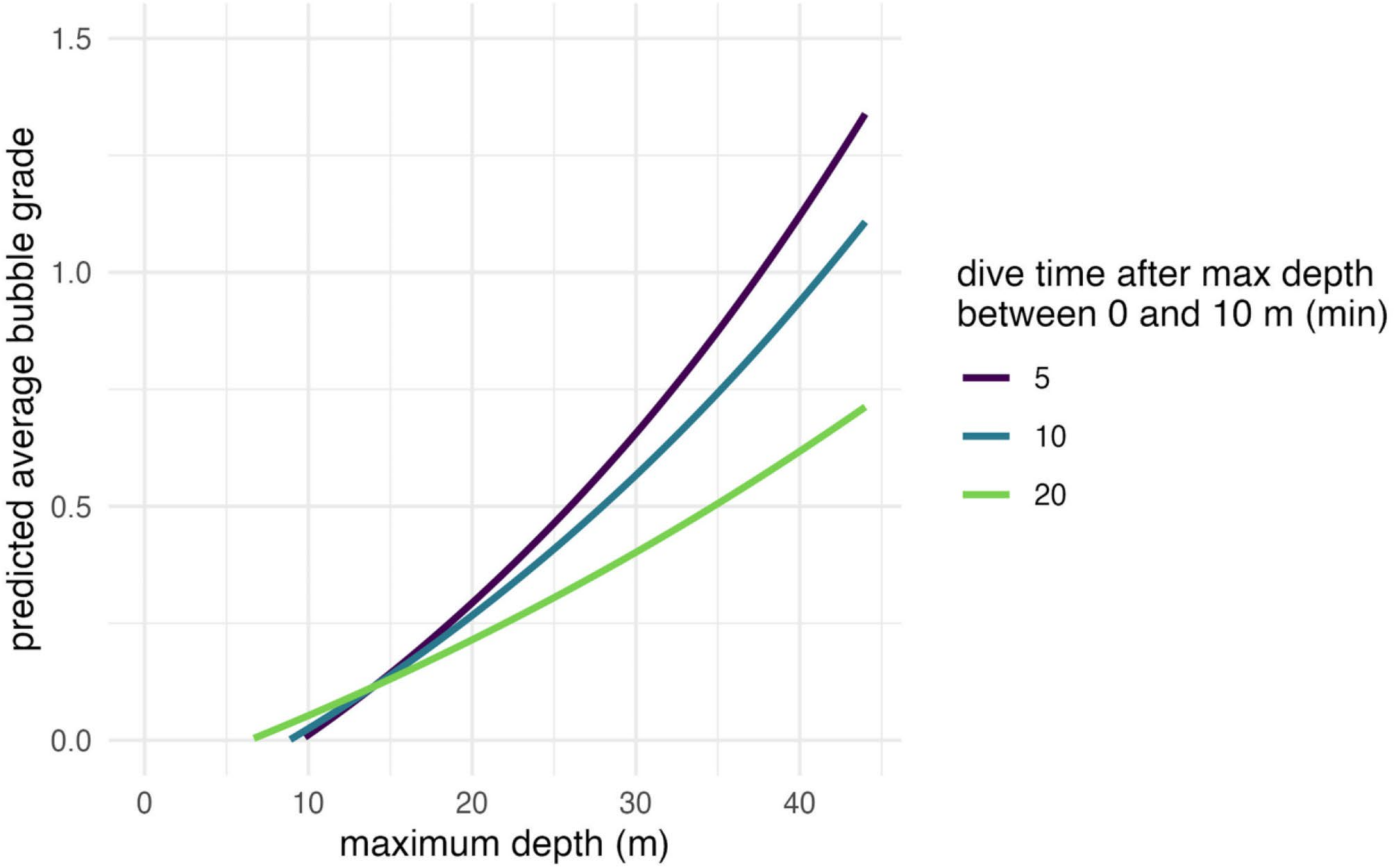
Predicted bubble grade using a linear model, showing that an extended period between 0 m and 10 m of depth drastically reduces the average bubble grade. Maximum depth exponentially increases the average bubble grade

Our analysis of the dive profiles (Fig. 3; Table 3) revealed that while maximum dive depth significantly predicted post-dive bubble grade (β = 0.41, *p* < 0.001), this effect was moderated by the time spent between 0 and 10 m depth during ascent. Specifically, the positive relationship between maximum depth and bubble grade weakened when divers spent more time in this shallow depth range after reaching maximum depth (interaction term β = -0.17, *p* < 0.001). This indicates that prolonged time spent ascending slowly through shallower depths can mitigate the increased risk of bubble formation associated with deeper dives. The model explained 17% of the variance in bubble grades (fixed effects). Individual differences derived from repeated measurements among divers accounted for 12%, which is a modest portion of the variance (intraclass correlation coefficient, ICC = 0.14).

**Table 3:** Results of a linear mixed model considering repeated measurements of 59 participants. The response variable, bubble grade 0–5 was log + 1 transformed. Beta and the standard error (SE) show the standardised regression coefficients of the predictors, describing the effect of the predictors in the model

Predictor	Regression coefficient	beta (SE)	t value	*p* value
intercept	-0.28489			
Maximum depth (m)	0.02787	0.41 (0.05)	8.28	< 0.001
Time between 0 and 10 m after max depth (min)	0.00868	-0.18 (0.05)	-3.47	< 0.001
interaction	-0.00067	-0.17 (0.05)	-3.71	< 0.001

Fixed effects R^2^ = 0.17, Random and fixed effects R^2^ = 0.29, 369 observations

### Predictive Modelling

For predictive modelling, Spearman Correlations of Predicted vs. Observed Bubble Grades were determined for the variable sets mentioned in Table 1. We assessed the performance of Random Forest models trained with the variable sets to predict bubble grades (Table 4). The models were trained on 70% of the data and tested on the remaining 30%.

**Table 4:** Correlation between echo-derived (observed) and predicted bubble grades. The “all grades” column shows the spearman correlation between the predicted and observed bubble grades across the entire test set. The “excluding 0 grades” column reports the spearman correlation after excluding test observations with a bubble grade of 0

Variable Set	Spearman Correlation *r*_s_ (All Grades)	Spearman Correlation *r*_s_ (Excluding 0 Grades)
Person-Specific Variables	0.08	0.10
Minimal Dive Data	0.44	0.34
Person-Specific + Dive Data	0.41	0.40
Person-Specific + Dive Data + Air	0.46	0.40
Dive Computer Data	0.49	0.40
All Variables	0.47	0.48

The Spearman correlation between observed and predicted bubble grades was used to evaluate the models. The model using dive computer data achieved the highest correlation (r_s_ = 0.49) across all grades, while the model with all variables closely followed with a correlation of r_s_ = 0.47. Models incorporating minimal dive data also performed well, with a correlation of r_s_ = 0.44.

When excluding observations with a bubble grade of 0, the model using all variables performed best with a Spearman correlation of 0.48, indicating strong predictive accuracy for higher bubble grades. The models using person-specific variables alone had the weakest performance, with correlations of r_s_ = 0.08 (all grades) and r_s_ = 0.10 (excluding grade 0), suggesting that dive-specific variables contribute significantly to improving model accuracy.

## Discussion

This study set out to (1) examine how both individual factors and dive profiles influence intravascular bubble formation in asymptomatic SCUBA divers, (2) validate and extend a previously proposed bubble-risk formula [8], and (3) contribute to improved guidelines for individualised risk estimation. Our findings support these aims by demonstrating that maximum dive depth was the strongest single predictor of post-dive bubble grade, showing a moderate positive relationship (r_s_ = 0.37). Air consumption and dive duration followed in importance, aligning with prior evidence that deeper and/or longer dives accumulate greater inert gas [7, 15, 16], but inert gas uptake and decompression stress are influenced by individual physical and psychological factors, thus showing the importance of air consumption as parameter combining individual and dive-related factors.

In validating the prediction formula by Fichtner et al. [8], we saw a modestly stronger correlation (r_s_ = 0.39) than the individual parameters alone—especially in predicting higher bubble grades. This underscores the practicality of combining depth, total time, age, and air consumption for a straightforward, individualised estimate of post-dive bubble formation. Notably, the formula requires minimal inputs, enabling divers to perform a basic risk stratification themselves, even without access to imaging technology. Despite multiple divers often following identical depth-time profiles when diving in a group, they can still end up with different bubble loads, a formula that accounts for personal characteristics is particularly relevant [2, 9].

When we incorporated additional dive computer data into mixed-effects and machine learning models, the predictions improved further, especially in identifying dives above bubble grade 0. Time spent in shallow water (0–10 m) after deep segments emerged as a key mitigating factor for bubble formation [1, 15]. This finding corroborates the longstanding recommendation for slow ascents and extended shallow stops [3, 16]. Our results showed that the model could distinguish not only between a bubble grade of 0 and grades above zero but also among the above-zero grades. The capacity to integrate these variables in near real-time suggests a future in which enhanced dive computers could provide personalised feedback about bubble risk as the diver ascends—a potential leap forward in avoiding decompression sickness [14].

In collecting data across two dive sites in freshwater and saltwater, we accounted for environmental differences that can sometimes influence buoyancy, temperature, and physiological stress [8, 10]. Although more volunteers initially expressed interest, only 59 completed the required measurements. Still, measuring in both environments strengthens external validity, as our findings are generally consistent across different conditions. This mirrors previous work showing that bubble formation patterns do not fundamentally differ by site, provided divers follow similar protocols [8].

Limitations must be acknowledged. First, although our sample size enabled meaningful analyses, it may limit the generalizability of results, especially regarding the wide variability of diver physiology. Second, a large proportion of dives yielded a bubble grade of 0, reflecting conservative diving and possibly constraining model performance in predicting the highest bubble grades. Third, the study did not directly control for diver experience level, though experience typically correlates with factors like air consumption [17]. Finally, our models rely on post-dive data, reducing immediate applicability. Integrating these formulas into dive computers for real-time feedback would be an essential next step [12]. Such an implementation would directly test whether alerting divers to their individualised risk prompts bubble-reducing measures (e.g., prolonged shallow stops, hydration) and decreases unprovoked diving incidents in broader populations [13].

## Conclusion

In conclusion, this study reaffirms that maximum depth is the leading predictor of post-dive bubble formation (r_s_ = 0.37), with air consumption and dive duration following closely. Our findings validate and slightly improve upon the previously proposed bubble-risk formula [8], which combines depth, age, surface interval, and air consumption. Notably, extended time in shallow water proved to be a simple yet effective measure for reducing bubble loads, underscoring the value of slow ascents and safety stops. These results highlight the potential for real-time integration of individualised risk assessments into dive computers, thereby enabling divers to adjust personal behaviour both during the ascent and throughout the surface interval—ultimately aiming to lower the risk of decompression sickness even when standard computer-derived profiles are strictly followed.

## Data Availability

An anonymised version of the dataset and all code to generate the analysis and plots is published on Zenodo (https://zenodo.org/records/15068564).
